# Supplementary material for: Formative Evaluation of a Home-Based Physical Activity Intervention for Adolescent Girls—The HERizon Project: A Randomised Controlled Trial
Source: Children (Basel). 2021 Jan 22;8(2):76. doi: 10.3390/children8020076 (PMC7911217; doi:10.3390/children8020076)
Supplement: Supplementary file 1 [file children-08-00076-s001.pdf]

Supplementary Material Supplementary Material

Table S1. COREQ (Consolidated criteria for Reporting Qualitative research) checklist

**No. Item**	**Guide questions/description**	**Reported on Page #**
**Domain 1: Research team and reflexivity**		
Personal Characteristics		
1. Interviewer/facilitator	Which author/s conducted the interview or focus group?	8
2. Credentials	What were the researcher's credentials? E.g. PhD, MD	8
3. Occupation	What was their occupation at the time of the study?	8
4. Gender	Was the researcher male or female?	8
5. Experience and training	What experience or training did the researcher have?	8
Relationship with participants		
6. Relationship established	Was a relationship established prior to study commencement?	8
7. Participant knowledge of the interviewer	What did the participants know about the researcher? e.g. personal goals, reasons for doing the research	8
8. Interviewer characteristics	What characteristics were reported about the interviewer/facilitator? e.g. Bias, assumptions, reasons and interests in the research topic	NA
**Domain 2: study design**		
Theoretical framework		
9. Methodological orientation and Theory	What methodological orientation was stated to underpin the study? e.g. grounded theory, discourse analysis, ethnography, phenomenology, content analysis	2
Participant selection		
10. Sampling	How were participants selected? e.g. purposive, convenience, consecutive, snowball	8
11. Method of approach	How were participants approached? e.g. face-to-face, telephone, mail, email	8
12. Sample size	How many participants were in the study?	9
13. Non-participation	How many people refused to participate or dropped out? Reasons?	9
Setting		
14. Setting of data collection	Where was the data collected? e.g. home, clinic, workplace	8
15. Presence of non-participants	Was anyone else present besides the participants and researchers?	NA
16. Description of sample	What are the important characteristics of the sample? e.g. demographic data, date	8
Data collection		
17. Interview guide	Were questions, prompts, guides provided by the authors? Was it pilot tested?	Supplementary material
18. Repeat interviews	Were repeat inter views carried out? If yes, how many?	N/A
19. Audio/visual recording	Did the research use audio or visual recording to collect the data?	9
20. Field notes	Were field notes made during and/or after the interview or focus group?	N/A
21. Duration	What was the duration of the inter views or focus group?	8
22. Data saturation	Was data saturation discussed?	N/A
23. Transcripts returned	Were transcripts returned to participants for comment and/or correction?	N/A
**Domain 3: analysis and findings**		
Data analysis		
24. Number of data coders	How many data coders coded the data?	9
25. Description of the coding tree	Did authors provide a description of the coding tree?	9
26. Derivation of themes	Were themes identified in advance or derived from the data?	9
27. Software	What software, if applicable, was used to manage the data?	9

28. Participant checking	Did participants provide feedback on the findings?	N/A
Reporting		
29. Quotations presented	Were participant quotations presented to illustrate the themes/findings? Was each quotation identified? e.g. participant number	14-15
30. Data and findings consistent	Was there consistency between the data presented and the findings?	14-15
31. Clarity of major themes	Were major themes clearly presented in the findings?	13-14
32. Clarity of minor themes	Is there a description of diverse cases or discussion of minor themes?	13-14

Table S2. The HERizon Project details: in accordance with the TIDieR checklist.

Item number	Item	Where is it located
**1**	**Brief name** Provide the name or phrase that describes the intervention.	1
**2**	**Why** Describe the rational, theory, or goal or the elements essential to the intervention.	1
**3**	**What** *Materials:* describe any physical or informational materials used in the intervention, including those provided to participants or used in intervention delivery or in training of intervention providers. Provide information on where the materials can be accessed (online appendix, URL).	13
**4**	*Procedures:* describe each of the procedures, activities, and/or processes used in the intervention, including any enabling or support activities.	6
**5**	**Who provided** For each category of intervention provider (e.g. psychologist, nursing assistance), describe their expertise, background and any specific training given.	5
**6**	**How** Describe the models of delivery (e.g. face-to-face or by some other mechanism, such as internet or telephone) of the intervention and whether it was provided individually or in a group.	5
**7**	**Where** Describe the type(s) of location(s) where the intervention occurred, including any necessary infrastructure or relevant features.	5
**8**	**When and how much** Describe the number of times the intervention was delivered and over what time period including the number of sessions, their schedule and their duration, intensity or dose.	5
**9**	**Tailoring** If the intervention was planned to be personalised, titrated or adapted, then describe what, why, when and how.	3
**10**	**Modifications:** If the intervention was modified during the course of the study, describe the changes (what, why, when and how).	NA
**11**	**How well** *Planned*: if intervention adherence or fidelity was assessed, describe how and by whom, and if any strategies were used to maintain or improve fidelity, describe them.	13
**12**	**How well** *Actual*: if intervention adherence or fidelity was assessed, describe the extent to which the intervention was delivered as planned.	13

Table S3. The 6-week individual behaviour change support call schedule for the intervention group.

Week	Topic
0	Familiarisation
1	Benefits of PA and why PA is important to them
2	Environmental restructuring
3	Social support
4	Free week[a]
5	Maintenance of PA and coping strategies
6	Celebration of achievements

Abbreviations: *PA physical activity*

All weeks included goal settings, action planning and barrier identification alongside the above week-specific topics.

[a]'Activity Mentors' were given freedom to discuss topics that were personally relevant to the participant in this week, e.g. body image.

Table S4. Interview guide of sample questions asked during semi-structure interviews.

Reach	How did you find out about taking part in the programme?

	What motivated you to take part?<br>How do you think we could get more girls to take part?
Implementation	Could you describe the programme for me? – for example if a friend asked you what taking part involved what would you say?
Adherence	How much did you stick to the 3 sessions provided?<br>• If adherence was high – what was it that helped them stick to it?<br>• If low – what were the reason? Did you do something else instead?<br>What did you think of the 3 sessions per week? How achievable was it for you? How much or little did it challenge you?<br>What do you think would increase the chances of you sticking to the programme? I.e. doing your 3 exercise sessions per week.
Impact	How do you think taking part in the programme has impacted you?<br>Are there any benefits or drawbacks about the programme?<br>How will you use what you have learned from the programme moving forwards?
Acceptability	Tell me your thoughts on the how the project was advertised, the consent process and the measures? i.e. fitness tests and questionnaires?<br>Can you tell me anything you have liked about the programme?<br>How could we make this programme more enjoyable for girls? Could you give me your opinion and feedback on what could be improved?
